# Supplementary material for: Sociocultural determinants of adoption of preventive practices for hantavirus: A knowledge, attitudes, and practices survey in Tonosí, Panama
Source: PLoS Negl Trop Dis. 2020 Feb 27;14(2):e0008111. doi: 10.1371/journal.pntd.0008111 (PMC7064252; doi:10.1371/journal.pntd.0008111)
Supplement: S1 Checklist — (DOC) [file pntd.0008111.s001.doc]

STROBE Statement—Checklist of items that should be included in reports of ***cross-sectional studies***

|  | Item No | Recommendation |
| --- | --- | --- |
| **Title and abstract** | 1 | (*a*) Indicate the study’s design with a commonly used term in the title or the abstract  Abstract in “Methodology and findings” Cross-sectional KAP survey |
|  |  | (*b*) Provide in the abstract an informative and balanced summary of what was done and what was found Abstract: Methodology and Findings |
| Introduction | | |
| Background/rationale | 2 | Explain the scientific background and rationale for the investigation being reported  Background, paragraphs 2,3, & 4 |
| Objectives | 3 | State specific objectives, including any prespecified hypotheses  Background, final paragraph |
| Methods | | |
| Study design | 4 | Present key elements of study design early in the paper  Methods, paragraph 1. “Cross-sectional community-based survey study” |
| Setting | 5 | Describe the setting, locations, and relevant dates, including periods of recruitment, exposure, follow-up, and data collection  Methods, paragraph 1. El Bebedero, Tonosi. March 2019. Data collection in seven neighborhoods by 5 field-interviewers. |
| Participants | 6 | (*a*) Give the eligibility criteria, and the sources and methods of selection of participants  Methods, paragraph 1 & 2. 18 years+, at least one year living in district. Door-to-door convenience sampling. |
| Variables | 7 | Clearly define all outcomes, exposures, predictors, potential confounders, and effect modifiers. Give diagnostic criteria, if applicable  Methods: “Survey instrument” section. |
| Data sources/ measurement | 8* | For each variable of interest, give sources of data and details of methods of assessment (measurement). Describe comparability of assessment methods if there is more than one group Methods: “Survey instrument” section. |
| Bias | 9 | Describe any efforts to address potential sources of bias Methods: “Survey instrument” section, paragraph 5. |
| Study size | 10 | Explain how the study size was arrived at  Methods, “Study setting, sample, recruitment” section, paragraph 1 |
| Quantitative variables | 11 | Explain how quantitative variables were handled in the analyses. If applicable, describe which groupings were chosen and why  Methods, “Data capture and analysis” section. |
| Statistical methods | 12 | (*a*) Describe all statistical methods, including those used to control for confounding Methods, “Data capture and analysis” section. |
|  |  | (*b*) Describe any methods used to examine subgroups and interactions n/a |
|  |  | (*c*) Explain how missing data were addressed Methods “Survey instrument” paragraph 3 |
|  |  | (*d*) If applicable, describe analytical methods taking account of sampling strategy n/a |
|  |  | (*e*) Describe any sensitivity analyses n/a |
| Results | | |
| Participants | 13* | (a) Report numbers of individuals at each stage of study—eg numbers potentially eligible, examined for eligibility, confirmed eligible, included in the study, completing follow-up, and analysed Results: “Sample characteristics” paragraph 1 |
|  |  | (b) Give reasons for non-participation at each stage n/a |
|  |  | (c) Consider use of a flow diagram |
| Descriptive data | 14* | (a) Give characteristics of study participants (eg demographic, clinical, social) and information on exposures and potential confounders Results: “Sample characteristics” paragraph 1 |
|  |  | (b) Indicate number of participants with missing data for each variable of interest  Results: “Predictors of preventive practices”, paragraph 1. |
| Outcome data | 15* | Report numbers of outcome events or summary measures Results, Table 4: Knowledge, practice, and HBM elements scores |
| Main results | 16 | (*a*) Give unadjusted estimates and, if applicable, confounder-adjusted estimates and their precision (eg, 95% confidence interval). Make clear which confounders were adjusted for and why they were included Results, Table 5 and Table 6 |
|  |  | (*b*) Report category boundaries when continuous variables were categorized N/A, for analysis, continuous variables were not categorized. |
|  |  | (*c*) If relevant, consider translating estimates of relative risk into absolute risk for a meaningful time period N/A |
| Other analyses | 17 | Report other analyses done—eg analyses of subgroups and interactions, and sensitivity analyses |
| Discussion | | |
| Key results | 18 | Summarise key results with reference to study objectives |
| Limitations | 19 | Discuss limitations of the study, taking into account sources of potential bias or imprecision. Discuss both direction and magnitude of any potential bias Discussion, final paragraph |
| Interpretation | 20 | Give a cautious overall interpretation of results considering objectives, limitations, multiplicity of analyses, results from similar studies, and other relevant evidence Conclusion paragraph |
| Generalisability | 21 | Discuss the generalisability (external validity) of the study results Discussion, final paragraph |
| Other information | | |
| Funding | 22 | Give the source of funding and the role of the funders for the present study and, if applicable, for the original study on which the present article is based Funding statement: The work was financed by the Department of Research in Emerging and Zoonotic Diseases and by the project 111130150.501 (BA) from the Ministry of Economy and Finance of Panama. CH was supported by a Fulbright grant from the Fulbright U.S. Student Program, sponsored by the United States Department of State. BA (DI-UIP63380000) is suppored by Research Direction, Universidad Interamericana de Panama. BA is a member of the SNI (Sistema Nacional de Investigación from SENACYT of Panamá). The funders had no role in study design, data collection and analysis, decision to publish, or preparation of the manuscript. |

*Give information separately for exposed and unexposed groups.

**Note:** An Explanation and Elaboration article discusses each checklist item and gives methodological background and published examples of transparent reporting. The STROBE checklist is best used in conjunction with this article (freely available on the Web sites of PLoS Medicine at http://www.plosmedicine.org/, Annals of Internal Medicine at http://www.annals.org/, and Epidemiology at http://www.epidem.com/). Information on the STROBE Initiative is available at www.strobe-statement.org.
