# Supplementary material for: Sociocultural determinants of adoption of preventive practices for hantavirus: A knowledge, attitudes, and practices survey in Tonosí, Panama
Source: PLoS Negl Trop Dis. 2020 Feb 27;14(2):e0008111. doi: 10.1371/journal.pntd.0008111 (PMC7064252; doi:10.1371/journal.pntd.0008111)
Supplement: S1 Data — Practice responses are not included here as they are provided in the main text. (DOCX) [file pntd.0008111.s002.docx]

S1 SurveyData: Frequency of responses for survey questions. Practice responses are not included here as they are provided in the main text.

**Knowledge questions**

How did you find out about hantavirus?

|  | N (124) | % |
| --- | --- | --- |
| Community (family, friends, neighbors, cases of hantavirus) | 91 | 73.4 |
| Television | 18 | 14.5 |
| Health care professionals | 18 | 14.5 |
| Radio | 7 | 5.6 |
| Newspaper | 4 | 3.2 |
| Other | 5 | 4 |
| Do not know/remember | 1 | 0.8 |

How is hantavirus transmitted to humans?

|  | N (124) | % |
| --- | --- | --- |
| Breathing air contaminated with mouse feces or urine (correct answer) | 75 | 60.5 |
| Referred to mouse (partially correct) | 13 | 10.5 |
| Do not know | 25 | 20.2 |
| Fumigations | 8 | 6.5 |
| Water | 1 | 0.8 |
| Food | 1 | 0.8 |
| Other | 10 | 8.1 |

What are the symptoms of hantavirus?

|  | N (124) | % |
| --- | --- | --- |
| Fever and chills | 97 | 78.2 |
| Headache | 73 | 58.9 |
| Pains | 49 | 39.5 |
| Difficulty breathing | 24 | 19.4 |
| Cough | 16 | 12.9 |
| Gastrointestinal symptoms | 14 | 11.3 |
| Sore throat | 6 | 4.8 |
| Others | 18 | 14.5 |
| Do not know | 17 | 13.7 |

What animals transmit hantavirus?

|  | N | % |
| --- | --- | --- |
| Mice | 107 | 86.3 |
| Others | 5 | 4 |
| Do not know | 12 | 9.7 |
| Caused by fumigation- not animal | 6 | 4.8 |

**Perceived susceptibility questions**

How worried are you about getting sick with hantavirus?

|  | Freq | % |
| --- | --- | --- |
| 1 Not worried | 27 | 21.8 |
| 2 A little worried | 13 | 10.5 |
| 3 Indifferent | 5 | 4.0 |
| 4 Worried | 41 | 33.1 |
| 5 Very worried | 37 | 29.8 |
| 99 Do not know | 1 | 0.8 |
| Total | 124 | 100.0 |

How worried are you that someone in your family will get sick with hantavirus?

|  | Freq | % |
| --- | --- | --- |
| 1 Not worried | 13 | 10.5 |
| 2 A little worried | 7 | 5.6 |
| 3 Indifferent | 4 | 3.2 |
| 4 Worried | 66 | 53.2 |
| 5 Very worried | 34 | 27.4 |
| Total | 124 | 100.0 |

How likely is it that over your lifetimes, yourself or someone in your family will get sick with hantavirus?

|  | Freq | % |
| --- | --- | --- |
| 1 Not likely | 12 | 9.7 |
| 2 Slightly likely | 14 | 11.3 |
| 3 Indifferent | 6 | 4.8 |
| 4 Likely | 50 | 40.3 |
| 5 Very likely | 19 | 15.3 |
| 99 Do not know | 23 | 18.5 |
| Total | 124 | 100.0 |

It is less likely that I will get sick with hantavirus in comparison to other people my age (Agree/disagree)

|  | Freq | % |
| --- | --- | --- |
| 2 Agree | 19 | 15.3 |
| 3 Indifferent | 14 | 11.3 |
| 4 Disagree | 48 | 38.7 |
| 5 Strongly disagree | 8 | 6.5 |
| 99 Do not know | 35 | 28.2 |
| Total | 124 | 100.0 |

I am healthy, so I cannot get contract hantavirus (Agree/Disagree)

|  | Freq | % |
| --- | --- | --- |
| 1 Strongly agree | 1 | 0.8 |
| 2 Agree | 23 | 18.5 |
| 3 Indifferent | 6 | 4.8 |
| 4 Disagree | 68 | 54.8 |
| 5 Strongly disagree | 11 | 8.9 |
| 99 Do not know | 15 | 12.1 |

Other groups in my community have a higher risk of getting sick with hantavirus than me. (Agree/disagree)

|  | Freq | % |
| --- | --- | --- |
| 1 Strongly agree | 4 | 3.2 |
| 2 Agree | 35 | 28.2 |
| 3 Indifferent | 11 | 8.9 |
| 4 Disagree | 40 | 32.3 |
| 5 Strongly disagree | 6 | 4.8 |
| 99 Do not know | 28 | 22.6 |
| Total | 124 | 100.0 |

**Perceived severity questions**

In your opinion, how dangerous is hantavirus?

|  | Freq | % |
| --- | --- | --- |
| 1 Not dangerous | 1 | .8 |
| 2 A bit dangerous | 2 | 1.6 |
| 3 Indifferent | 1 | .8 |
| 4 Dangerous | 55 | 44.4 |
| 5 Very Dangerous | 62 | 50.0 |
| 99 Do not know | 3 | 2.4 |
| Total | 124 | 100.0 |

If you got sick with hantavirus, how severe do you think the illness would be?

|  | Freq | % |
| --- | --- | --- |
| 1 Not severe | 1 | .8 |
| 2 A bit severe | 13 | 10.5 |
| 3 Indifferent | 4 | 3.2 |
| 4 Severe | 31 | 25.0 |
| 5 Very severe | 37 | 29.8 |
| 99 Do not know | 38 | 30.6 |
| Total | 124 | 100.0 |

If you got sick with hantavirus, how likely is it that you would die from the illness?

|  | Freq | % |
| --- | --- | --- |
| 1 It is not probable | 6 | 4.8 |
| 2 A little probable | 11 | 8.9 |
| 3 Indifferent | 4 | 3.2 |
| 4 Probable | 38 | 30.6 |
| 5 Very probable | 21 | 16.9 |
| 99 Do not know | 44 | 35.5 |
| Total | 124 | 100.0 |

In your opinion, how easy or difficult is it to recover from hantavirus?

|  | Freq | % |
| --- | --- | --- |
| 2 Easy | 5 | 4.0 |
| 3 Indifferent | 5 | 4.0 |
| 4 Difficult | 74 | 59.7 |
| 5 Very Difficult | 23 | 18.5 |
| 99 Do not know | 17 | 13.7 |
| Total | 124 | 100.0 |

If I got sick with hantavirus, I would still be able to work (Agree/Disagree)

|  | Freq | % |
| --- | --- | --- |
| 1 Strongly agree | 5 | 4.0 |
| 2 Agree | 28 | 22.6 |
| 3 Indifferent | 5 | 4.0 |
| 4 Disagree | 67 | 54.0 |
| 5 Strongly disagree | 5 | 4.0 |
| 99 Do not know | 14 | 11.3 |
| Total | 124 | 100.0 |

If I got sick with hantavirus, I would lose a lot of money (Agree/Disagree).

|  | Freq | % |
| --- | --- | --- |
| 1 Strongly disagree | 3 | 2.4 |
| 2 Disagree | 18 | 14.5 |
| 3 Indifferent | 13 | 10.5 |
| 4 Agree | 76 | 61.3 |
| 5 Strongly agree | 6 | 4.8 |
| 99 Do not know | 8 | 6.5 |
| Total | 124 | 100.0 |

**Perceived benefits questions:**

In your opinion, how effective are the following actions for the prevention of hantavirus?

|  | Not effective | A Little effective | In different | Effective | Very effective |
| --- | --- | --- | --- | --- | --- |
|  | Freq (%)   N=124 | Freq (%)   N=124 | Freq (%)   N=124 | Freq (%)   N=124 | Freq (%)   N=124 |
| Take out trash/keeping patio clean and orderly | 4 (3.2) | 11 (8.9) | 2 (1.6) | 97 (78.2) | 10 (8.1) |
| Eliminate accumulation of waste materials and move accumulations of wood/forages/bales a minimum of 30 meters away from the home | 3 (2.4) | 12 (9.7) | 1 (0.8) | 100 (80.6) | 8 (6.5) |
| Cut grass and weeds around the home | 5 (4) | 9 (7.3) | 0 | 104 (83.9) | 6 (4.8) |
| Set traps for mice and rats | 11 (8.9) | 9 (7.3) | 7 (5.6) | 93 (75) | 4 (3.2) |
| Use mask, handkerchief, other to cover your mouth and nose when cleaning | 5 (4) | 6 (4.8) | 0 | 105 (84.7) | 8 (6.5) |
| Use rubber gloves when cleaning | 7 (5.6) | 8 (6.5) | 4 (3.2) | 101 (81.5) | 4 (3.2) |
| Wet surfaces with water or disinfectant before sweeping/cleaning | 2 (1.6) | 4 (3.2) | 1 (0.8) | 109 (87.9) | 8 (6.5) |
| Keep trash in closed containers or tanks resistant to rodents | 1 (0.8) | 4 (3.2) | 0 | 113 (91.1) | 6 (4.8) |
| Keep grains in airtight or sealed containers | 1 (0.8) | 3 (2.4) | 0 | 113 (91.1) | 6 (4.8) |
| Seal holes/cracks | 3 (2.4) | 4 (3.2) | 0 | 113 (91.1) | 4 (3.2) |
| Keep food and water in containers with a lid, out of reach of mice. | 0 | 3 (2.4) | 1 (0.8) | 116 (93.5) | 4 (3.2) |
| Ventilate closed bodegas, depositories, or small spaces for a minimum of 30 min before entering | 2 (1.6) | 6 (4.8) | 4 (3.2) | 109 (87.9) | 3 (2.4) |

If I adopt all the recommended prevention measures to prevent hantavirus, I would not get sick.

|  | Freq | % |
| --- | --- | --- |
| 1 Strongly disagree | 5 | 4.0 |
| 2 Disagree | 24 | 19.4 |
| 3 Indifferent | 5 | 4.0 |
| 4 Agree | 70 | 56.5 |
| 5 Strongly agree | 5 | 4.0 |
| 99 Do not know | 15 | 12.1 |
| Total | 124 | 100.0 |

**Perceived obstacles**

How difficult or easy is it for you to carry out the following actions?

|  | Muy fácil | Fácil | Indiferente | Difícil | Muy dificil |
| --- | --- | --- | --- | --- | --- |
|  | Freq (%)   N=124 | Freq (%)   N=124 | Freq (%)   N=124 | Freq (%)   N=124 | Freq (%)   N=124 |
| Take out trash/keeping patio clean and orderly | 2 (1.6) | 90 (72.6) | 2 (1.6) | 30 (24.2) | 0 |
| Eliminate accumulation of waste materials and move accumulations of wood/forages/bales a minimum of 30 meters away from the home | 0 | 101 (81.5) | 4 (3.2) | 19 (15.3) | 0 |
| Cut grass and weeds around the home | 0 | 113 (91.1) | 5 (4) | 6 (4.8) | 0 |
| Set traps for mice and rats | 3 (2.4) | 105 (84.7) | 6 (4.8) | 9 (7.3) | 1 (0.8) |
| Use mask, handkerchief, other to cover your mouth and nose when cleaning | 2 (1.6) | 112 (90.3) | 5 (4) | 5 (4) | 0 |
| Use rubber gloves when cleaning | 1 (0.8) | 99 (79.8) | 10 (8.1) | 14 (11.3) | 0 |
| Wet surfaces with water or disinfectant before sweeping/cleaning | 4 (3.2) | 115 (92.7) | 3 (2.4) | 2 (1.6) | 0 |
| Keep trash in closed containers or tanks resistant to rodents | 1 (0.8) | 119 (96) | 0 | 4 (3.2) | 0 |
| Keep grains in airtight or sealed containers | 1 (0.8) | 121 (97.6) | 1 (0.8) | 1 (0.8) | 0 |
| Seal holes/cracks | 1 (0.8) | 107 (86.3) | 8 (6.5) | 7 (5.6) | 1 (0.8) |
| Keep food and water in containers with a lid, out of reach of mice. | 2 (1.6) | 120 (96.8) | 1 (0.8) | 1 (0.8) | 0 |
| Ventilate closed bodegas, depositories, or small spaces for a minimum of 30 min before entering | 1 (0.8) | 121 (97.6) | 1 (0.8) | 1 (0.8) | 0 |

**Perceived obstacles (continued)**

Each time a participant responded “difficult” or “very difficult” to an action, we asked “why?”. Summaries of responses are given below for actions which 10% or more of participants answered “difficult” or “very difficult”.

Taking out trash and keeping house/patio clean and orderly

| Obstacles | Freq | % |
| --- | --- | --- |
| Money | 1 | 3.3 |
| Resources/materials | 2 | 6.7 |
| Time | 5 | 16.7 |
| Do not feel like it | 1 | 3.3 |
| Physical restrictions (time, age, health) | 14 | 46.7 |
| Others | 7 | 23.3 |
| Total | 30 | 100.0 |

Eliminating accumulations of waste materials/move accumulates of wood/forages/bales a minimum of 30 meters for the home

|  | | |
| --- | --- | --- |
| Obstacles | Freq | % |
| Money | 1 | 5.3 |
| Resources/materials | 3 | 15.8 |
| Time | 3 | 15.8 |
| Do not feel like it | 1 | 5.3 |
| Physical restrictions (time, age, health) | 5 | 26.3 |
| Others | 6 | 31.6 |
| Total | 19 | 100.0 |

Using rubber gloves when cleaning

| Obstacles | Freq | % |
| --- | --- | --- |
| Money | 4 | 28.6 |
| Resources/materials | 3 | 21.4 |
| Time | 0 | 0.0 |
| Do not feel like it | 0 | 0.0 |
| Uncomfortable | 4 | 28.6 |
| Other | 3 | 21.4 |
| Total | 14 | 100.0 |

**Perceived obstacles (continued)**

In your opinion, why do people in your community not adopt the recommended prevention measures?

|  | Freq | % (n=124) |
| --- | --- | --- |
| Not enough money | 1 | 0.8 |
| Do not have time | 2 | 1.6 |
| They do not feel like it | 38 | 30.6 |
| They do not think the practices will work | 8 | 6.5 |
| Lack of information/knowledge | 7 | 5.6 |
| Carelessness | 12 | 9.7 |
| They do not believe in hantavirus/do not believe it is caused by the mouse/believe fumigations are to blame | 35 | 28.2 |
| Do not know | 18 | 14.5 |
| Others | 25 | 20.2 |

**Cues to action questions**

| N=124 | Yes (%) | No (%) |
| --- | --- | --- |
| People in my community talk about the prevention measures against hantavirus. | 81 (65.3) | 43 (34.7) |
| Other residents in my community remind me to carry out the prevention measures against hantavirus. | 64 (51.6) | 60 (48.4) |
| Leaders in my community (government authorities, doctors, etc) remind me to carry out the prevention measures | 83 (66.9) | 41 (33.1) |
| I have heard or seen messages about hantavirus and the prevention measures through television radio, flyers, or others | 114 (91.9) | 10 (8.1) |

**Information sources questions**

Do you know here you could get information about hantavirus?

|  | Freq. | % (n=124) |
| --- | --- | --- |
| 1 Yes | 85 | 68.5 |
| 2 No | 39 | 31.5 |

If so, where?

|  | Freq | % (n=85) |
| --- | --- | --- |
| 2 Television | 1 | 1.2 |
| 3 Radio | 1 | 1.2 |
| 4 Health center/hospital | 67 | 78.8 |
| 5 Internet | 4 | 4.7 |
| 7 Other | 4 | 4.7 |
| 8 Community | 8 | 9.4 |

Would you like to learn more about hantavirus?

|  | Freq | % (n=124) |
| --- | --- | --- |
| Yes | 115 | 92.7 |
| No | 9 | 7.3 |

If so, through which medium would you prefer to receive Hantavirus information?

|  | Freq | % |
| --- | --- | --- |
| 1 Newspaper | 3 | 2.4 |
| 2 Television | 29 | 23.4 |
| 3 Radio | 5 | 4.0 |
| 4 Health centers/health professionals | 22 | 17.7 |
| 5 Home visits | 29 | 23.4 |
| 6 Internet | 1 | .8 |
| 8 Phone messages (WhatsApp, etc) | 2 | 1.6 |
| 10 Do not know | 2 | 1.6 |
| 11 No preference | 8 | 6.5 |
| 12 Talks/meetings | 6 | 4.8 |
| 13 Written mediums/flyers | 8 | 6.5 |

From whom do you trust to receive health information?

|  | Freq | % (n=124) |
| --- | --- | --- |
| 1 Health professionals/health centers | 107 | 86.3 |
| 2 Family or Friends | 1 | .8 |
| 4 TV or radio | 5 | 4.0 |
| 5 Other | 3 | 2.4 |
| 99 Do not know | 8 | 6.5 |
