# Supplementary material for: Sociocultural determinants of adoption of preventive practices for hantavirus: A knowledge, attitudes, and practices survey in Tonosí, Panama
Source: PLoS Negl Trop Dis. 2020 Feb 27;14(2):e0008111. doi: 10.1371/journal.pntd.0008111 (PMC7064252; doi:10.1371/journal.pntd.0008111)
Supplement: Attachment — Submitted filename: Peer Review for PLOS Neglected Tropical Diseases - pntd-d-19-01059.docx [file pntd.0008111.s003.docx]

Peer Review for PLOS Neglected Tropical Diseases

**Sociocultural determinants of adoption of preventive practices for hantavirus: A knowledge, attitudes, and practices survey in Tonosí, Panama (ID: PNTD-D-19-01059)**

**Methods**

The authors need to explicitly state that the seven neighborhoods in the study were purposefully selected based on their large population size; meaning that smaller neighborhoods were excluded from the study. Therefore the results are biased toward the larger communities and not representative of El Bebedero. It is important to state this upfront because it has implications for how the results are interpreted. Because households and individuals were also conveniently selected, then it’s evident that random selection did not occur at any level. I’m curious to know how the authors went from neighborhood selection to household selection? Did data collection teams go to every household in each neighborhood to give them the chance to be included in the survey? Or was an intermediary step used (i.e. selection of clusters within neighborhoods) ?

I ask these questions, because when doing a KAP survey, or any survey for that matter, it is very important to be able to know who the findings are ‘valid’ for (i.e. external validity). In this case, your results may only be potentially applicable to just those respondents in the 7 neighborhoods whose households were conveniently selected and had someone at home at the time of the visit who was willing to provide consent. As you can see there are many caveats here in terms of who the findings may be applied to.

The sample size calculation assumptions described by the authors are based on having a sampling frame of all individuals in Bebedero and using simple random sampling to select individuals. The sample size obtained from this is usually called the effective sample size (ESS) or the minimum required sample when using simple random sampling. Apparently simple random sampling was not their sampling approach used so therefore the sample size calculations are incorrect because they ignore other important parameters. At a minimum, they needed to have accounted for the intraclass correlation (ICC) which would have undoubtedly increased the ESS because the design effect would likely have been at least 2 or more; meaning that ESS of 95 needed to be multiplied by at least 2, which would’ve meant about 200 respondents plus inflation for non-response (200 divided by response rate; e.g. 200/.80 = 250). In sum, it would have been more appropriate to just use multi-stage cluster sampling, and then account for the cluster design using inverse probability weights for each level of selection. State this somewhere in the limitation section.

The section on consent is a bit confusing. Consent was sought after residents agreed to participate? (lines 164-165: If a resident agreed to participate in the survey, they read the informed consent and were allowed to ask questions). I’m assuming this was a translation/language issue. However, need to review and be sure to have it reflect what was done. Normal procedure is to explain the purpose of the study, its risks, benefits, etc (consent statement) then seek informed consent from the participant.

Because of the limitations posed by the sampling, the authors need to recognize that they are limited in the type of analysis that can be conducted without violating underlying assumptions of the statistical tests used. Therefore, while I generally appreciate the use of regression models, I think that the sample was not powered appropriately for the hypothesis testing. Therefore, my recommendation is for the authors to focus on the descriptive results (those currently included in the supplemental material) with the understanding that the results are not generalizable but formative.

The regression models are inappropriate for use in this study, and any conclusions drawn from them would be misleading because the standard errors are under-estimated by not accounting for the intraclass correlation (which violates the assumption of independence in regression modeling). In essence the statistical software is ‘thinking’ that the sample was obtained from simple random sampling from a population-based sampling frame (which obviously is not the case) when determining significance in testing the hypotheses. Therefore, the null may have been rejected when it shouldn’t have because of the of the incorrect standard errors. So things that are significant are either (1) not significant or (2) the sample size is not large enough to know if they are in fact significant. Therefore, the most the authors can do is to describe results using proportions and aggregated mean KAP scores, etc. Because of the limited evidence available on the topic they have presented on, the descriptive results still have good public health value. In a way, the authors should view this as an early exploratory assessment (formative research) to inform future KAP surveys on hantavirus that will use more rigorous sampling approaches and complex analyses in the same population or similar populations elsewhere.

The big take-away here is that when conducting a KAP survey, the sampling approach determines how the analysis can be conducted. Fancy analytical approaches cannot override sub-par sampling techniques and lack of statistical power. Convenience sampling should only be done when a random sample is impossible to obtain due to specific characteristics of the population being targeted (e.g. hidden population without good sampling frame), or when wanting to just get some quick/rapid ‘impressions/insights’ to inform future work (the current paper falls in this category in my opinion and should be framed as such). They authors should not shy away from what they’ve done but rather learn from it and embrace its limitations as a stepping stone in the right direction for their future work and that of others in their field.

**Results**

As mentioned in my comments regarding the methods, the analysis need to focus on describing the results using what they already have included in the supplementary material. However, it won’t be realistic to include all the supplementary l tables. Instead the authors must try to find ways to combine the tables and/or summarize the results a bit more and keep just the key tables and leave the rest as suppl.

**Conclusions**

The conclusion would need to be revised based on revisions to the presentation of the results. Generally, the conclusions for this assessment should be formative – i.e. trying to inform future areas of work and/or focus regarding assessments of hantavirus infection prevention at the community level in the absence of biomedical treatment and vaccines.

**Editorial and data presentation modifications**

Major revisions needed regarding presentation of the results.

**Summary and general comments**

The authors have addressed an important research area focusing on the sociocultural determinants of hantavirus prevention practices and knowledge among community members in a highly affected geographic area in Panama prone to the hantavirus infections. Given that biomedical treatments and vaccines are not available to tackle this pathogen, community-based prevention practices are critical for disease control. Therefore, this KAP study address an issue with public health importance for a specific population and potential implications to inform other studies elsewhere.

In the background, instead of saying “we predicted” it’s better to say “hypothesized” because you predict after collecting data and hypothesized before that.

It’s also better to say that the Health Belief Model informed the design of the KAP instead of saying that the survey was based on HBM. Basing the survey on HBM will require psychometric validation of the HBM constructs used in the study, which to my understanding was not done. Also, note that cues-to-action is not an attitude and should not be described as such. Self-efficacy is an important construct in HMB but missing from this study. How confident people are in their ability to execute the behavior is strong predictor of the likelihood of behavior uptake. The authors therefore need to explain the omission of self-efficacy in their use of HBM. In addition, there are many other individual health behavior models, why HBM? Need to justify the use of HBM in the context of the alternatives. Using theory to ground a KAP survey requires a lot of steps that are currently missing. Therefore, the authors may want to de-emphasize HBM as a grounding theory and more so as a theoretical guidance coupled with the prior literature on KAPs on hantavirus. In the background, a more synthesized summary of HBM should be sufficient, and then in the methods the authors can elaborate on the specific adaptations for their study.

The regression models are inappropriate for use in this study, and any conclusions drawn from them would be misleading because the standard errors were not corrected to account for the intraclass correlation. However, there are some really interesting results in the supplementary material that can be synthesized nicely in the results section to tell an important story about the potential barriers and enablers of hantavirus prevention practices in this highly affected population.

**Confidential comments to editor**

I would recommend accepting this paper with major revisions, mainly in how the results are presented. The regression models are inappropriate because the standard errors are under-estimated due to the sampling approach used by the authors. Nevertheless, the descriptive results they’ve provided as supplementary materials should be further synthesized and incorporated into the results – with the focus on key enablers and barriers to behavior uptake. Consistent with my feedback in the methods, the authors should frame this paper as an early exploratory assessment of KAP to inform future assessments using more rigorous approaches. I think there is scientific merit here worth publishing if they authors can reframe their results, embrace the limitations, and discuss how the results can inform future research on uptake of behaviors to prevent hantavirus infections in high risk populations.
